# Asymptomatic Apical Hypertrophic Cardiomyopathy Uncovered During Routine Preoperative Evaluation

**DOI:** 10.7759/cureus.89362

**Published:** 2025-08-04

**Authors:** Emily Strickland, Takor B Arrey-Mbi

**Affiliations:** 1 Internal Medicine, Madigan Army Medical Center, Tacoma, USA; 2 Medicine/Cardiology, Madigan Army Medical Center, Tacoma, USA

**Keywords:** abnormal ekg, apical hypertrophic cardiomyopathy, cardiac sudden death, ekg abnormalities, hypertrophic cardiomyopathy (hcm), left ventricular hypertrophy (lvh), preoperative evaluation, preoperative planning

## Abstract

Apical hypertrophic cardiomyopathy (ApHCM) is an uncommon, nonobstructive form of hypertrophic cardiomyopathy (HCM) that is associated with an increased risk of ventricular aneurysms, atrial fibrillation, heart failure, and cardiac death. In this case report, a 63-year-old male patient was found to have deeply negative T waves on electrocardiogram (EKG) during a routine preoperative evaluation in an outpatient internal medicine clinic. Imaging with echocardiography and cardiac magnetic resonance confirmed the diagnosis of ApHCM. Once the diagnosis was made, the patient underwent a thorough, guideline-directed evaluation for risk of sudden cardiac death (SCD), including ambulatory cardiac monitoring, cardiac MRI, and genetic testing. The patient continues to receive longitudinal care, including medical therapies with a beta-blocker, and periodic follow-up assessments of SCD risk.

## Introduction

Hypertrophic cardiomyopathy (HCM) represents a heterogeneous disease that results in hypertrophy of the left ventricle (LV) in the absence of another condition capable of producing such a magnitude of hypertrophy. HCM most commonly affects the basal anterior septum and the anterior free wall; however, several other HCM patterns have been described [1]. Apical hypertrophic cardiomyopathy (ApHCM) is a rare pattern of HCM that accounts for around 3% of HCM in the United States [2]. This pattern of HCM involves the distal LV and is classically associated with "giant" negative precordial T waves on electrocardiography and "spade-like" appearance on echocardiography. Due to its effects on the LV below the level of the papillary muscles, ApHCM is not associated with left ventricular outflow tract (LVOT) obstruction that is seen in classical HCM [3]. 

## Case presentation

**Figure 1:**
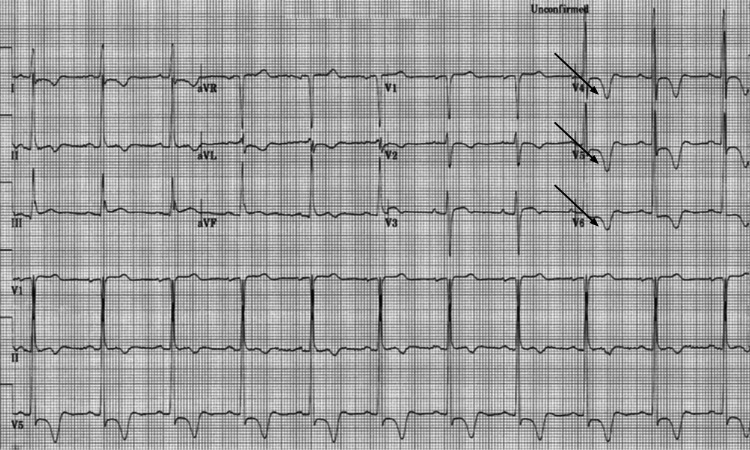
EKG showing left ventricular hypertrophy with deep negative T waves in precordial leads (indicated by arrows) with maximal negative deflection of 11 mm. EKG: Electrocardiogram

**Figure 2:**
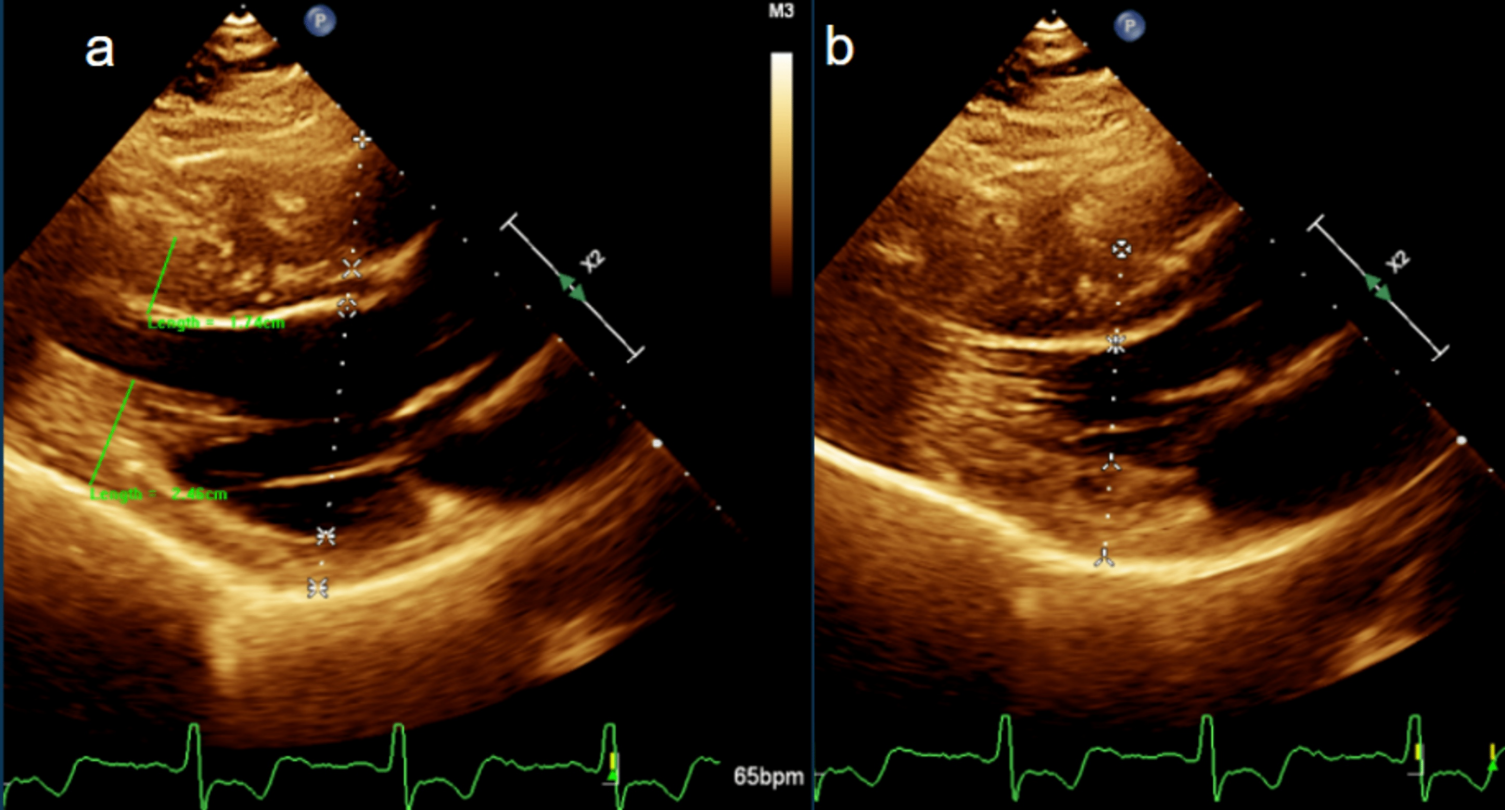
ApHCM transthoracic echocardiography parasternal long axis view in diastole (a) and in systole (b) showing maximal wall thickness of 17.4 mm. ApHCM: Apical hypertrophic cardiomyopathy

**Figure 3:**
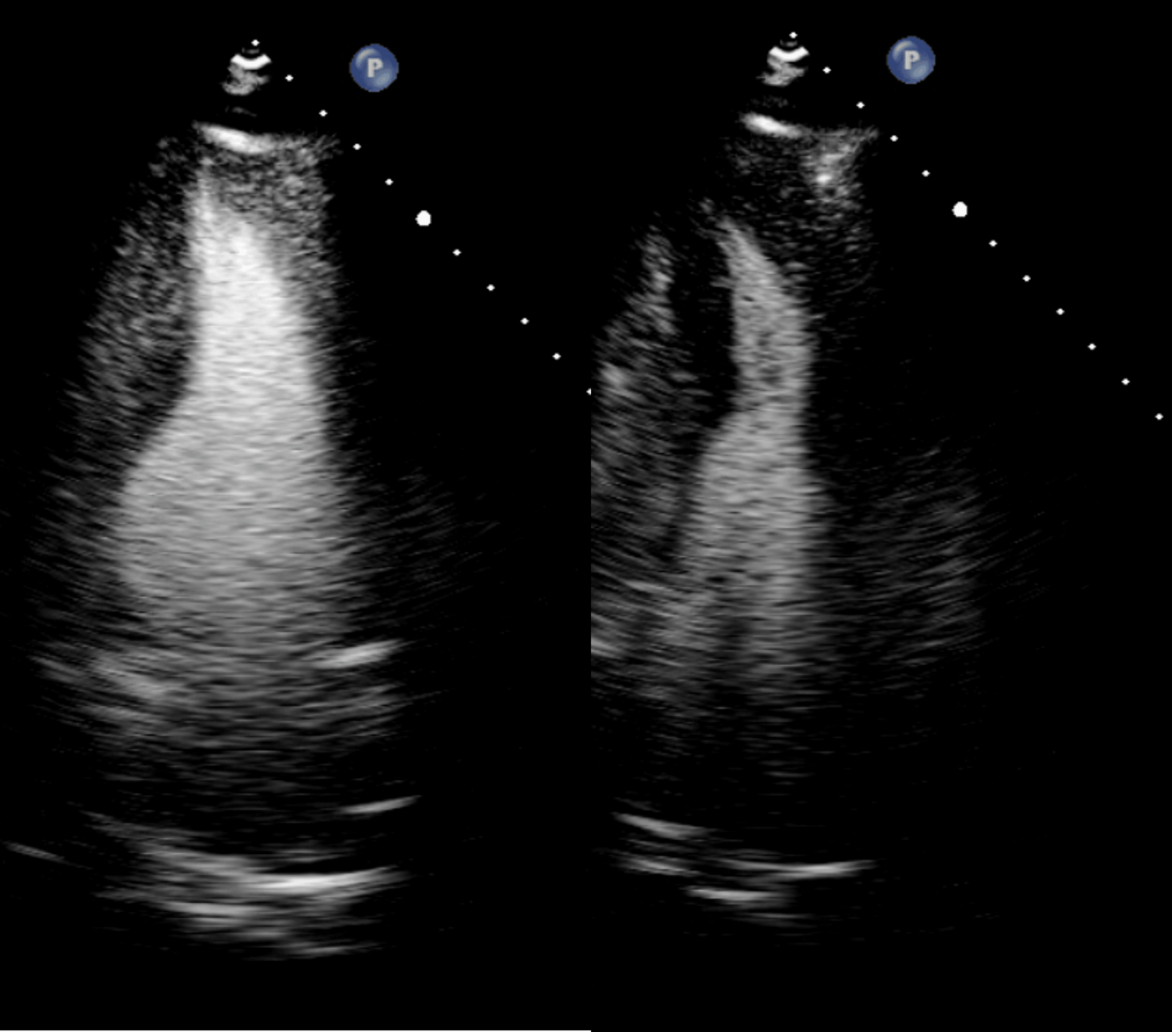
ApHCM TTE apical 2 chamber in diastole (a) and in systole (b) showing “spade-like” LV appearance. ApHCM: Apical hypertrophic cardiomyopathy; TTE: Transthoracic echocardiogram; LV: Left ventricle

A 63-year-old male patient with a medical history significant for type 2 diabetes mellitus, essential hypertension, and spinal stenosis with neurogenic claudication presented to an outpatient internal medicine clinic for preoperative evaluation prior to an elective C3-C6 cervical laminoplasty. He was in his usual state of health and denied any chest pain, palpitations, dyspnea, orthopnea, peripheral edema, lightheadedness, or syncope. Electrocardiogram (EKG) revealed normal sinus rhythm with left ventricular hypertrophy and deep negative T waves with negative deflection up to 11 mm in precordial leads, suggestive of ApHCM (Figure 1). Serial EKG and labs, including a complete metabolic panel, were unremarkable. In the absence of symptoms, these EKG changes were considered unlikely to be ischemic. Transthoracic echocardiography revealed a left ventricular ejection fraction (EF) of greater than 65% and thickening of the apical left ventricular wall (Figures 2-3). After this evaluation, the patient was ultimately cleared by cardiology for surgery.

Four days later, the laminoplasty procedure was aborted during anesthesia induction due to reported worsening of baseline ST depressions seen on telemetry. Due to concern for non-ST elevation myocardial infarction (NSTEMI), the patient was evaluated by the inpatient cardiology team. High-sensitivity troponin was mildly elevated but stable. The patient denied any symptoms suggestive of acute coronary syndrome. He was discharged with a cardiac MRI and cardiac ambulatory monitoring ordered. Cardiac magnetic resonance confirmed left ventricular wall thickening in the apex with maximum wall thickness of 26 mm as well as areas of mid-myocardial late gadolinium enhancement appearing to involve less than 10% of total myocardial mass (Figure 3). Fourteen-day ambulatory cardiac monitoring showed one asymptomatic, nine-beat run of monomorphic ventricular tachycardia at a rate of 139 bpm. The patient was started on a beta-blocker and referred to a genetic counselor. The patient had no family history of sudden cardiac death (SCD) or arrhythmias within three generations. Genetic testing with a standard HCM genetic panel did not reveal a genetic cause of his disease. With all of this information, his risk of SCD was assessed with the assistance of the ESC-HCM Risk Calculator (European Society of Cardiology, France). He was determined to be at low risk for SCD, and placement of an implantable cardioverter defibrillator (ICD) was deferred.

## Discussion

ApHCM is an uncommon form of HCM that may be found in asymptomatic individuals during routine evaluation. It is characterized by “giant” negative precordial T waves on EKG, defined as negative voltage of greater than 1 mV [2] and “spade-like” appearance of the LV [4]. Diagnosis is confirmed when imaging shows apical wall thickness of >15 mm; however, cases may be missed with echocardiography, and use of echo contrast or cardiac magnetic resonance may be necessary to confirm diagnosis in patients with suspected ApHCM. Although once thought of as a benign HCM pattern, hypertrophy of the apex can lead to myocardial ischemia and left ventricular cavity obliteration [4]. This myocardial ischemia and left ventricular cavity obliteration can lead to chest pain, dyspnea on exertion, and elevated cardiac biomarkers, and also increase the risk of developing ventricular aneurysms, atrial fibrillation, and heart failure. More recent data suggest that ApHCM is associated with increased cardiac death rates of 0.5% to 4% annually, which approaches that of other forms of HCM [5]. 

Once the diagnosis is made, ApHCM management focuses on control of symptoms and mitigating the risk of SCD. Beta-blockers and nondihydropyridine calcium channel blockers (CCB) are recommended by the American College of Cardiology guidelines for control of symptoms related to ApHCM; however, the benefit in asymptomatic patients remains unclear. The American College of Cardiology guidelines recommend assessing the risk of cardiac arrest at diagnosis and then every one to two years [1]. This risk assessment should include a detailed review of the personal and family history of cardiac arrest and arrhythmias. Imaging should be conducted to determine LV thickness and EF and evaluate for ventricular aneurysms. Ambulatory cardiac monitoring is needed to detect any arrhythmias. In addition, it is important to monitor and treat comorbid conditions such as coronary artery disease, obesity, hypertension, and sleep disorders, as these comorbidities can worsen the effects of HCM [1].

## Conclusions

In conclusion, this is an uncommon case of ApHCM found incidentally in an asymptomatic middle-aged man undergoing preoperative evaluation. Diagnosis relied on recognizing the unique EKG pattern of "giant" negative precordial T waves and confirming the findings with multimodality imaging, which showed wall thickness of >15 mm. Once the diagnosis was made, the patient underwent a thorough work-up to evaluate the risk of SCD and continues to receive periodic re-evaluation, as the risk for SCD may change over time. Ultimately, this case highlights the importance of recognizing findings that are associated with increased risk of cardiac death during routine evaluations of asymptomatic individuals.
